# Quality assessment of primary health care for infants and young children in Hunan Province of China

**DOI:** 10.1186/s12875-026-03516-x

**Published:** 2026-08-10

**Authors:** Tao Li, Shuaiming Zhang, Ni Jia, Yaohua Dai

**Affiliations:** 1 Department of Child Health Big Data Research Center, Capital Center for Children’s Health, Capital Medical University, Capital Institute of Pediatrics, Beijing, 100020 China; 2 Department of Clinical Nutrition, Capital Center for Children’s Health, Capital Medical University, Capital Institute of Pediatrics, Beijing, 100020 China; 3 Department of Integrated Early Childhood Development, Capital Center for Children’s Health, Capital Medical University, Capital Institute of Pediatrics, Beijing, 100020 China

**Keywords:** Primary health care, Infants, Children, Healthcare facilities, Facility assessment, China

## Abstract

**Objective:**

To comprehensively evaluate the service quality of primary health care (PHC) for children aged 0–3 years in healthcare facilities of China, and to identify strengths and existing gaps to provide evidence for optimizing regional child health service policies.

**Methods:**

A multi-stage stratified random sampling method was used to select 35 healthcare facilities from 4 geographical regions in Hunan province of China. Data were collected using the WHO Western Pacific Regional Office Primary Health Care Quality Improvement Guide, which included facility assessments, medical record reviews, and caregiver interviews. All indicators were assessed against China national basic public health service standards and WHO PHC quality benchmarks. Chi-square test was used for comparative analysis between sick and healthy children.

**Results:**

The overall PHC foundation was sound: 85.0% of service rooms had qualified handwashing facilities, 92.0% were equipped with alcohol rub, and over 97% of facilities had standardized protocols for breastfeeding screening and infection control. The key basic medicines and routine growth monitoring supplies achieved an availability rate above 91.4%. The under-6-month exclusive breastfeeding rate was 38.9%, and National Immunization Programme (NIP) vaccine coverage remained over 90% for most routine vaccines. However, significant gaps existed. Well children showed far higher health screening rates than sick children, with growth measurement completion rates of 93.9% vs. 72.2% for weight and 92.1% vs. 9.0% for height; respiratory rate screening remained extremely low at around 17% in both groups. Non-NIP vaccine coverage was only 28.5%–47.1%. The availability of therapeutic food, special vaccines and essential emergency medicines was below 30%, and only 45.8% of rooms were equipped with sharps containers and merely 28.6% of facilities had child abuse and neglect management protocols. Additionally, the staff IMCI training rate was only 54.2%, and patient–staff ratios ranged from 48.6% to 65.9% across clinics.

**Conclusion:**

Child PHC services in Hunan Province perform well in basic infrastructure, routine health management and national immunization, but have prominent deficiencies in standardized clinical assessment for sick children, breastfeeding promotion, non-NIP vaccine accessibility, child protection system construction, essential drug supply, and human resource allocation. Targeted interventions on clinical standardization, material guarantee, professional training and child protection mechanism improvement are needed to further elevate the overall quality and equity of early childhood PHC services.

**Supplementary Information:**

The online version contains supplementary material available at https://doi.org/10.1186/s12875-026-03516-x.

## Introduction

Over the past three decades, China has achieved remarkable progress in child health: the under-five mortality rate (U5MR) declined from 39.7‰ in 2000 to 7.1‰ in 2021, neonatal mortality rate (NMR) decreased from 22.8‰ to 3.1‰ [1]. By comparison, the global U5MR fell from 76‰ to 38‰ and the NMR from 31‰ to 18‰ over the same period. China is far below the global average and also lower than the median for upper‑middle‑income countries [2]. Even so, there were still roughly 65,700 under-five deaths in 2022 in China, among which 45.1% took place during the neonatal period [3]. Early childhood is a critical window period for physical growth and cognitive development, and the quality of primary health care (PHC) for children aged 0–3 years directly determines children’s short-term health outcomes and long-term life health quality, which is also a core indicator of national public health service capacity [4]. As an important part of the national basic public health service system, child PHC focuses on standardized health management, disease prevention and early development of young children, and plays a pivotal role in reducing U5MR, improving children’s growth and development status, and promoting early childhood development (ECD). It follows a regular schedule of health check-ups across different age groups: home visits and examinations in the neonatal period, eight routine visits for children aged 1–36 months, and annual assessments for preschoolers aged 4–6 years. Each visit includes standardized evaluations on growth, physical condition, neurodevelopment, anemia, hearing and vision, alongside immunization management and health guidance on feeding, disease and injury prevention [5]. In recent years, China has continuously improved the construction of the PHC service system and issued a series of standardized management guidelines for children’s health to enhance the quality and coverage of PHC services for children [6–9]. These policies advocate for integrated service delivery models, improved workforce training, and the establishment of standardized care protocols to ensure consistent service quality nationwide. Despite these policy frameworks, challenges remain in translating policy into practice effectively. The actual impact on service quality, coverage, and sustainability requires rigorous evaluation.

This study evaluated the overall quality of PHC services for children aged 0–3 years using standardized and verified assessment tools combining on-site observation, medical record review and caregiver interviews. Based on the empirical data, this study systematically analyzed the current situation and key influencing factors of PHC service quality for children aged 0–3 years, as well as the differences in service performance between sick and healthy children, aiming to provide targeted data support and practical optimization suggestions for improving the quality of regional child PHC services.

## Methods

### Study design and setting

This cross-sectional study was conducted in Hunan Province, China, from March to June 2021. Hunan was selected due to its demographic size and regional representativeness. Hunan Province, with a 2018 permanent population of 69 million and an under-5 mortality rate of 5.34‰, is representative of China’s central region in terms of socioeconomic development and health service distribution. The study covered 4 geographical and socioeconomic regions (eastern, western, southern, northern) to ensure sample diversity. Inclusion criteria for healthcare facilities were: (1) providing PHC for children aged 0–3 years; (2) public-owned (to ensure alignment with national PHC policies); and (3) operational during the study period.

### Study population

Inclusion criteria for children were: (1) aged 0–3 years; (2) accompanied by at least one primary caregiver who could communicate in Mandarin or Hunan dialect; and (3) no history of severe chronic diseases (e.g., congenital heart disease) that might affect service assessment. Exclusion criteria for children were: Children with incomplete medical records or caregivers who refused to participate in the interview. Well children were defined as those who attended the facility solely for routine health check-ups, while all other visiting children were categorized as sick children. This study was approved by the Ethics Committee of the Capital Institute of Pediatrics (SHERLL2021027). The written informed consents were obtained from child’s parent.

### Sampling method

A four-stage stratified random sampling approach was used to select study facilities, ensuring representation across all PHC levels:

1. Provincial-level facilities: 3 facilities (Hunan Provincial People’s Hospital, Hunan Provincial Maternal and Child Health Hospital, Hunan Children’s Hospital) were included, as they are the top-tier providers of child health services in the province.
2. Prefecture-level facilities: The 14 prefectures in Hunan were divided into 4 regions (east, west, south, north) based on geography and per capita GDP (Gross Domestic Product). One prefecture was randomly selected per region, with 2 facilities (a general hospital and a maternal and child health hospital) included per prefecture (total: 8 facilities).
3. County-level facilities: In each sampled prefecture, counties were categorized as “close” (within the first half of distance to the prefecture center) or “distant” (second half). One county was randomly selected per category, with 2 facilities per county (total: 16 facilities).
4. Township-level facilities: In each sampled county, one medium-distance township (based on distance to the county center) was selected, with 1 facility per township (total: 8 facilities).

At each facility, 5 sick and 5 well children were enrolled.

### Assessment tool

The assessment tool was adapted from the WHO Western Pacific Regional Office Primary Health Care Quality Improvement Guide (Module 3: Care for Infants and Young Children) [10]. The tool included three validated components:

1. Facility checklist: Evaluated infrastructure (e.g., handwashing facilities, waste management), drug/supply availability (e.g., antibiotics, growth charts), and policy protocols (e.g., IMCI (Integrated Management of Childhood Illness) implementation, child protection).
2. Child clinical checklist: Collected data on vital sign recording (temperature, respiratory rate), growth monitoring (weight, height), feeding practices, illness management (diarrhea, respiratory illness), and vaccine recording.
3. Caregiver interview checklist: Assessed breastfeeding difficulties, injury prevention knowledge and ECD concerns.

The tool’s reliability was tested in a pre-survey of 30 children (Cronbach’s α = 0.87), confirming internal consistency.

### Data collection and quality control

Twelve evaluators (including national, provincial, and district child health experts) were trained for 2 days on tool use, interview techniques, and data recording. Evaluators were divided into 4 groups (3 members each), with at least one member per group trained in IMCI and local dialect proficiency. Data were collected via: (1) on-site observation of outpatient services; (2) review of medical records; (3) face-to-face interviews with caregivers (conducted in a private room to ensure confidentiality). Informed verbal consent was obtained from all caregivers. Data were double-entered into an online system, with 10% of records verified for accuracy.

### Statistical analysis

Data were cleaned and analyzed using R (version 4.1.2). Categorical variables were presented as frequencies and percentages (n, %). Stratified analyses were performed to compare key indicators across child health status (sick vs. well) using the chi-square test for categorical variables. A two-sided *P*-value < 0.05 was considered statistically significant.

## Results

### Characteristics of the sample

The proportion of children aged 0–3 years ranged from 1.78% to 3.53% of the total population across the four sampled prefectures. The inclusion rate of facilities varied by level. All provincial facilities providing pediatric services in Hunan were included. For municipal facilities, the inclusion rate was 2/3 in Chenzhou, which has a standalone municipal children’s hospital, and 100% in the remaining three cities. The overall inclusion rates stood at 25% for county-level facilities and 5.8% for township health centers. A total of 312 (89.1%) children aged 0–36 months from 35 facilities were included. The age distribution was relatively even across four groups. Sick children accounted for 46.6% of the sample, with 42.8% being female and 57.2% male. Most children were recruited from county-level facilities (47.8%), followed by prefecture-level (24.0%) and township-level (18.9%) facilities, while 9.3% were from provincial-level facilities. The characteristics of the children are shown in Table 1.

**Table 1:** Characteristics of the sampled children (*n* = 312)

Characteristics		Percentage (%)
Age (months)	0–5	27.2
	6–11	19.9
	12–23	28.5
	24–36	24.4
Sex	Female	42.8
Child status	Sick	46.6
Facility level	Provincial	9.3
	Prefecture	24.0
	County	47.8
	Township	18.9

### Clinical practice quality

#### Basic health screening

**Fig. 1:**
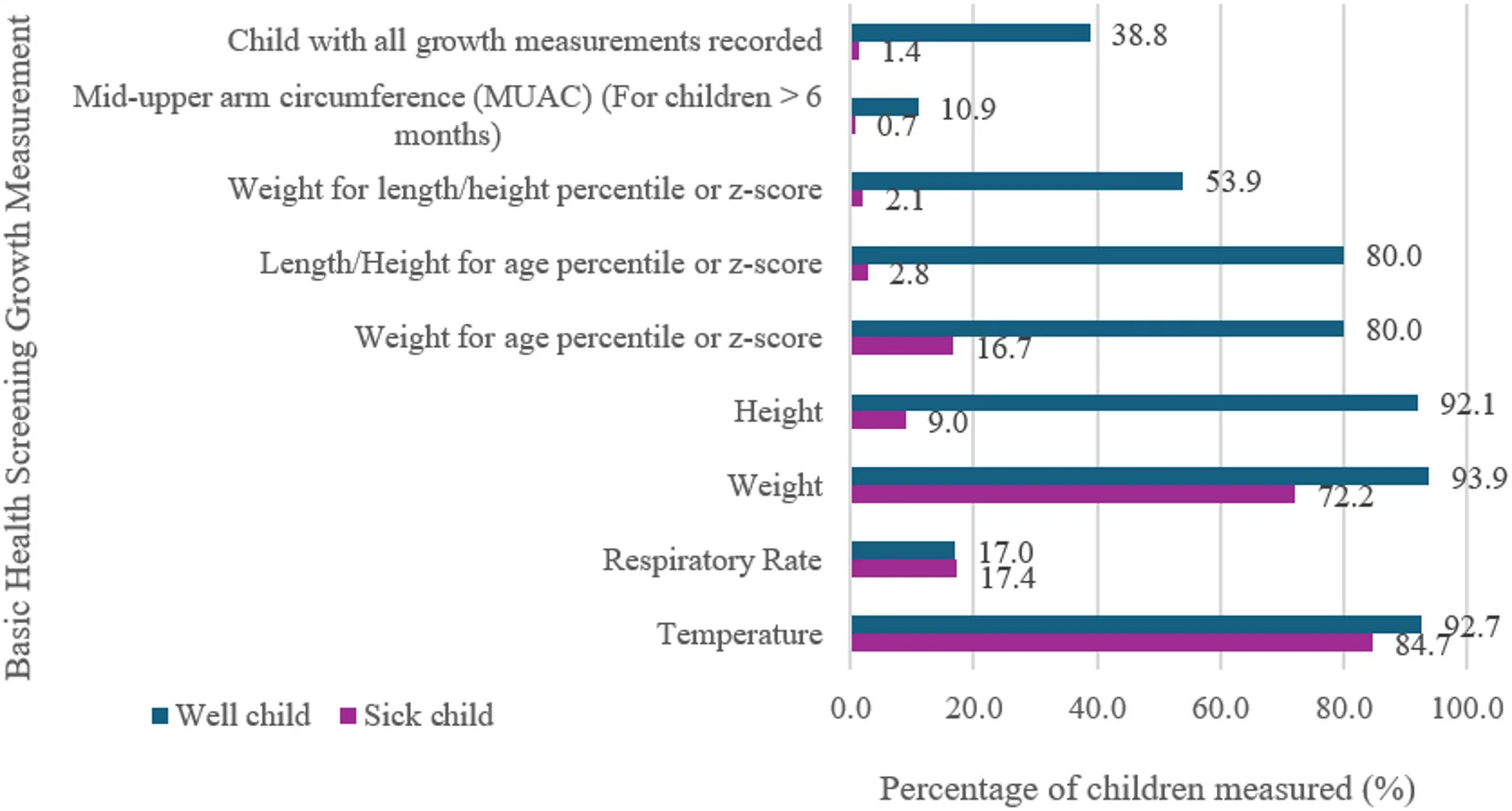
Basic health screening rates by child status

The highest screening rates were observed for basic anthropometric measures in well children, including weight (93.9%), height (92.1%), and length/height-for-age z-score/percentile (80.0%), while sick children had markedly lower rates for these items (*P* < 0.001 for all). Weight-for-length/height z-score/percentile was recorded in 53.9% of well children but only 2.1% of sick children. For vital sign assessment, temperature screening was high in both groups, though still significantly higher in well children compared to sick children ((92.7% vs. 84.7%, *P* < 0.05); respiratory rate screening was extremely low in both groups, with no significant difference between well and sick children (17.0% vs. 17.4%) (Fig. 1).

#### Infant and young child feeding

##### Breastfeeding

34.7% of children in the assessment were being breastfed, and 28.7% of the interviewed mothers reported they had breastfeeding difficulties. The exclusive breastfeeding rate for children under 6 months was 38.9%. Among children under 6 months, 27.8% received breastfeeding screening, and 82.0% of caregivers received breastfeeding counseling.

##### Complementary feeding

For children over 6 months, 68.0% consumed at least 4 food types daily, and 23.0% of caregivers reported feeding difficulties or concerns. Complementary feeding screening was provided to 59.5% of these children, and 74.3% of caregivers received feeding advice or counselling on how to feed their child properly from health workers. Additionally, 91.7% of children over 6 months had ever received vitamin A (or mixture of vitamin A and D), and 77.1% received vitamin A in the last 6 months from the date of the assessment.

#### Illness management

##### Diarrhea

Of the 27 children with diarrhea, 96.3% of caregivers were queried about diarrhea duration, while 51.9% were asked about the presence of bloody diarrhea. All 3 children with bloody diarrhea received antibiotics, whereas only 12.5% of those with watery diarrhea were given oral rehydration solution (ORS), zinc, and advice on increased fluid intake, and none received multivitamins or antibiotics.

##### Respiratory illness

Of the 87 children with respiratory illness, 40.2% presented with wheezing. Among these, 94.3% received a trial of inhaled bronchodilator or nebulizer treatment from health workers, yet only 25.7% were provided with a home inhaler for a 5-day trial or advised to return for nebulizer use. 45.5% of caregivers were given counselling or advice to reduce indoor air pollution including smoking, 10.3% were advised to change cooking fuel or cook outside the house, and 40.7% were advised to improve household ventilation.

##### Fever

Of the 52 children with fever, 98.1% of caregivers were asked about fever duration. Of the 41 children received antibiotics, 75.6% of their caregivers were explained why antibiotics were given, and only 23.1% of caregivers were informed about follow-up criteria (in how many days or in which situation the caregivers should bring the child back).

#### Vaccination

**Fig. 2:**
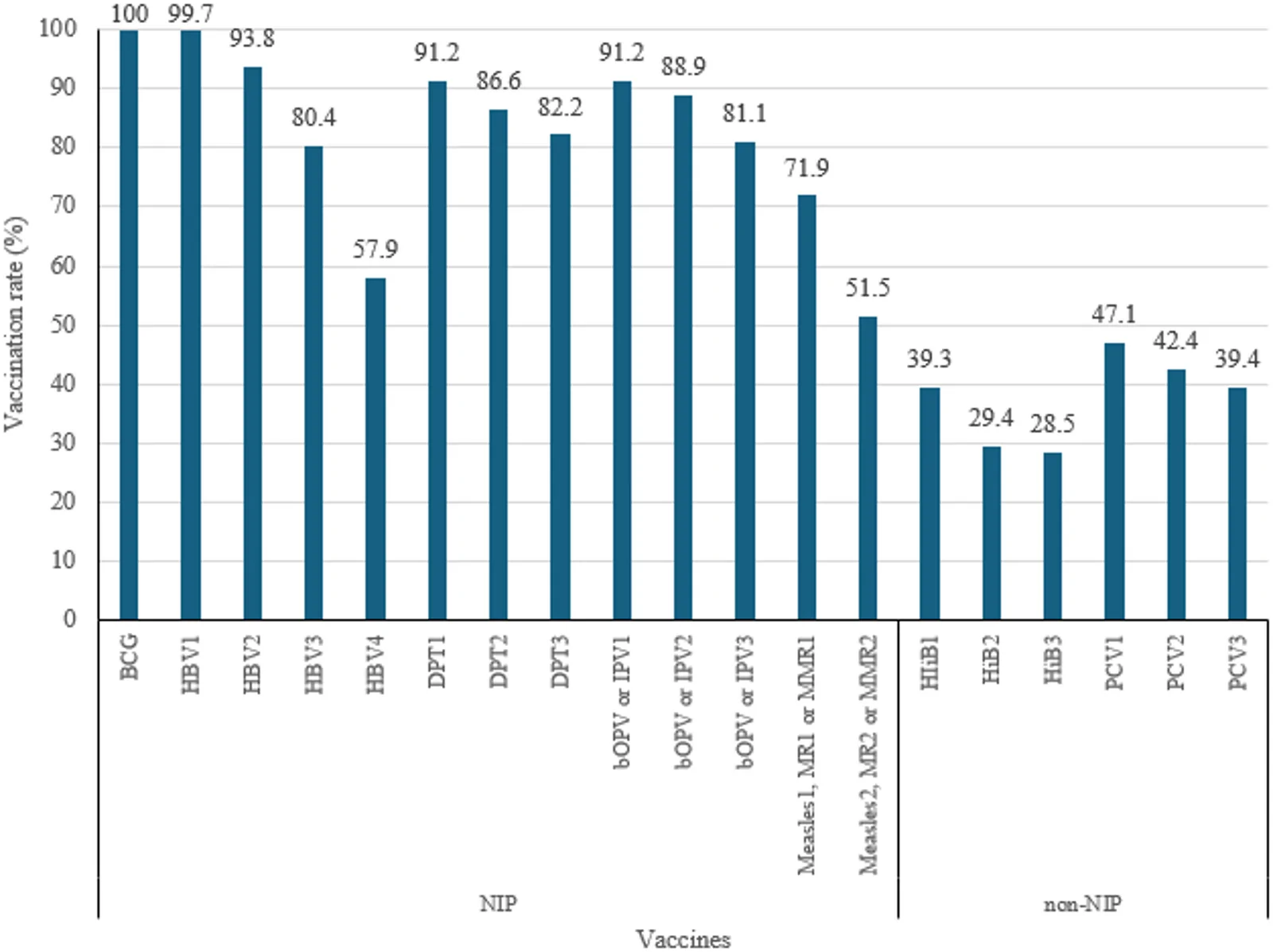
Vaccination rate of children. *NIP: National Immunization Programme; BCG: Bacille Calmette-Guérin Vaccine༛HBV: Hepatitis B Vaccine༛DPT: Diphtheria-Tetanus-Pertussis Vaccine༛bOPV: bivalent Oral Poliovirus Vaccine༛IPV: Inactivated Poliovirus Vaccine༛MMR: Measles, Mumps, Rubella Vaccine༛MR: Measles-Rubella Vaccine༛Hib: Haemophilus influenzae type b Vaccine༛PCV: Pneumococcal Conjugate Vaccine

Coverage rates for National Immunization Programme (NIP) vaccines were generally high, with the first-dose rates remaining above 90% for most core vaccines (Fig. 2). Bacille Calmette-Guérin Vaccine (BCG ) and the first dose of Hepatitis B Vaccine (HBV) achieved perfect coverage, at 100.0% and 99.7%, respectively. The coverage of HBV remained high across doses, with HBV2 at 93.8% and HBV3 at 80.4%. The fourth dose (HBV4) showed the lowest coverage among NIP vaccines at 57.9%, which is designated for infants born to hepatitis B antigen-positive mothers. The coverage of Diphtheria-Tetanus-Pertussis Vaccine (DPT) remained consistent across doses, with DPT1 at 91.2%, DPT2 at 86.6%, and DPT3 at 82.2%, showing a gradual decline with subsequent doses. The coverage of bivalent Oral Poliovirus Vaccine/Inactivated Poliovirus Vaccine (bOPV/IPV) was stable, ranging from 88.9% to 91.2% across the three doses. The first dose of Measles or Measles-Mumps-Rubella Vaccine (MMR) reached 71.9%, while the second dose (Measles2/MMR2) dropped to 51.5%, representing the lowest coverage within the NIP category.

In contrast, coverage rates for non-NIP vaccines were substantially lower, typically ranging from 28.5% to 47.1%. Haemophilus influenzae type b (Hib) vaccine’s coverage was low across all three doses, with Hib1 at 39.3%, Hib2 at 29.4%, and Hib3 (the final dose) at 28.5%. Similar to Hib, Pneumococcal Conjugate Vaccine (PCV) coverage was moderate but low, with PCV1 at 47.1%, PCV2 at 42.4%, and PCV3 at 39.4%.

#### Child protection and ECD

Thirteen children had injuries requiring medical attention. Correspondingly, 61.5% of the cause of the injury was contusion, and 23.1% was burn. 69.2% of caregivers received injury prevention counseling, and 57.1% could name three or more age-appropriate prevention methods.

Only 9.7% of caregivers were asked about ECD concerns, but 93.3% of those with concerns were referred for further assessment. 48.1% of caregivers could name three or more age-appropriate strategies for promoting child development. Most parents had already been doing it but didn’t know it was a measure or strategy, and if they faced the list of all the items, they could choose out more appropriate strategies than they could give the accurate names of the measures.

### Facility assessment

#### Infrastructure

85.0% of 679 rooms had adequate handwashing facilities (clean, have continuous clean and running water, have soap available, and have single-use towels or hand dryers available), and 92.0% of them had alcohol gel/hand rub. Among 609 client toilets, 97.9% were functional/clean, 14.1% had menstrual hygiene facilities, and 36.8% were wheelchair-accessible. 45.8% of 679 rooms had sharps containers, 62.3% had medical waste containers, and 29.6% had general waste containers.

#### Medicine and supply availability

Basic supplies were widely available. Glucose solution, weighing scales, and antibiotics for pneumonia management were the most widely available items, with rates reaching 97.1%, 97.1% and 94.3%. Similarly, normal saline IV, vitamin K, and growth charts had high availability (91.4%). Therapeutic food formulations (RUTF), micronutrient powders, and vaccines including pentavalent and measles vaccines were available in less than one-third of facilities. Supplies such as zinc drops, eye ointments, and syringes for vaccination showed moderate availability (around 60%–65%). In contrast, several critical commodities showed notably low availability. Antiretroviral (ARV) and co-trimoxazole for Human Immunodeficiency Virus (HIV)-related care, as well as injectable gentamicin for infant sepsis, had the lowest rates (8.6%–11.4%). Details see in Figure S1.

#### Policies

97.1% of facilities had breastfeeding screening protocols, and 88.6% had ECD screening protocols. For sick child outpatient policies, over 90% of the facilities had written policies and staff oriented on the policies in areas such as routine health screening, routine breastfeeding screening for infants 0–24 months (exclusive breastfeeding < 6 months), and clinical case-definition of anemia (Figure S2). For infection control practices including processing contaminated instruments and disinfection of equipment including stethoscopes and thermometers between patients, over 97% of the facilities had written policies and staff oriented on the policies (Figure S3). For protocol for management and referral of children with suspicion of abuse or maltreatment at home, 28.6% of facilities had the policy (Figure S4). 60.0% of facilities had Integrated Management of Newborn and Child Illness (IMNCI) standards adopted and used in outpatient clinics (Figure S5).

Patient staff ratios for well-child clinics, sick-child clinics, vaccination clinics, and outpatient treatment rooms were respectively 62.9%, 62.9%, 48.6%, 65.7%.

For staff continuous education situation, total of 54.2% facility staffs was trained for IMCI. 97.1% of staff received training on Kangaroo Mother Care (KMC) / Infant and Young Child Feeding (IYCF) / Nutrition topics. 99.9% of staff received training on HIV/TB (Tuberculosis) topics. 89.1% of staff received training on ECD/Childhood Disability topics. 80.2% of staff received training on Injuries, abuse, maltreatment topics (Figure S6).

## Discussion

This study systematically assessed outpatient child PHC services across different facility levels in Hunan province of China. Hunan’s PHC demonstrated solid foundations: well-equipped infection control facilities, sufficient basic medical supplies, high coverage of NIP vaccines, widespread standardized service protocols, and extensive staff training on child nutrition and injury prevention. Routine growth monitoring for well children was adequately delivered, with timely referrals for early childhood development concerns. However, major service gaps existed. Sick children had far lower completion rates of growth and nutritional assessments than well children; respiratory rate screening was rare for all children. Few diarrheal children received standard ORS and zinc treatment. Non-NIP vaccine coverage remained low. Few facilities stocked therapeutic food and emergency medicines, few had child maltreatment protocols, only half staffs completed IMCI training, and exclusive breastfeeding lagged behind national goals.

The high basic anthropometric measures rate, high temperature recording rate and increasing exclusive breastfeeding rate [11] reflect the effectiveness of China’s PHC policies [8]. Additionally, the high coverage of free vaccines underscores the success of China’s Expanded Programme on Immunization (EPI), launched in 1978 to provide equitable access to routine vaccines [12, 13]. The high referral rate for caregivers with ECD concerns demonstrates that facilities prioritize follow-up for children with developmental issues, even if proactive ECD screening is limited. This aligns with global recommendations to address ECD gaps through targeted referrals [14].

The government’s push for standardized care is reflected in the high prevalence of formal protocols; nearly all facilities had written policies for breastfeeding screening, routine health assessment, infection prevention and control, and anemia management, and most had ECD screening policies. Furthermore, staff training coverage was high for nutrition, HIV/TB prevention, ECD, and injury/abuse prevention, demonstrating successful efforts to build workforce capacity. The robust foundational conditions observed in this study reflect the tangible outcomes of China’s long-term commitment to improving child health [15, 16]. Since 2009, the Chinese government has integrated child health management into the National Essential Public Health Service Program, providing free and standardized services for children aged 0–6 years across urban and rural areas. This policy support is evident in our findings: basic infrastructure for infection control was generally strong, with most rooms equipped with adequate handwashing facilities and alcohol-based hand rub, and nearly all client toilets were functional and clean.

Meanwhile, this study also identified several gaps. Overall, well children consistently demonstrated substantially higher screening rates than sick children for nearly all basic health screening items. This phenomenon may be explained by the fact that basic health monitoring, growth and development assessment, and health counseling are well implemented in child routine health check-ups, but are frequently neglected when delivering medical services to sick children [17]. Even for the measurement of vital signs, like respiratory rate, no higher assessment rates were observed among sick children compared with well children. Sign assessment for sick children is frequently overlooked in outpatient settings, especially in facilities lacking full IMCI-trained workforce [18]. Moreover, the weight-for-height z-score and percentile, one of key indicators for malnutrition screening, yielded a low screening coverage rate. Even among well children, only half received this assessment. This problem needs to be prioritized in future practice, for instance, by reinforcing relevant requirements in official guidance documents and assessment indicators.

The exclusive breastfeeding rate for infant under 6 months in this study was higher than report of China Development Research Foundation in 2019 (29.2%) [11], but was still below the national target of 50% by 2025 [9] and the global average [19]. This low rate reflects significant gaps in early childhood nutrition and primary health care service delivery, highlighting an urgent need for targeted interventions to improve feeding practices and caregiver support. Standardized diarrhea management with ORS and zinc remains a global service gap. Cross-national facility surveys across Central Asian and sub-Saharan low- and middle-income countries revealed that less than half of sick children with watery diarrhea received combined ORS and zinc intervention, consistent with our local findings [18]. WHO’s IMCI implementation guidelines emphasize universal provision of these two core medicines for childhood diarrhea, thus regular professional training is suggested for medical staff to standardize clinical medication and ensure standardized management of childhood diarrhea.

The low coverage of self-funded vaccines is consistent with national trends, and acceptance, awareness and affordability emerged as the most notable themes [20]. It is critical to boost non-NIP vaccine coverage and eliminate regional inequities in vaccine access. Stakeholders across government, public and society should work together to resolve supply and demand constraints found in the research, guaranteeing equal access to essential vaccines across the country. Several measures can be adopted to improve the low uptake of non-NIP vaccines. Targeted health education should be delivered to eliminate misunderstandings among parents and raise their awareness of the value of non-NIP vaccination. Primary care facilities need to optimize service arrangements and standardize vaccination practices, while ensuring stable vaccine supply. Policymakers may consider gradually incorporating self-paid vaccines into the NIP. Furthermore, reminding eligible children of vaccination during routine child health services and launching catch-up programs through multi-sector cooperation will effectively increase overall vaccination coverage.

The low proportion of facilities with child abuse/neglect protocols reflects insufficient attention from healthcare workers and a lack of policy prioritization for child protection. Actually, child maltreatment is common in China [21]. Traditional Chinese parenting norms, which emphasize “strict discipline,” may contribute to underreporting of abuse and limited institutional response [22]. Sustained efforts should be made to ensure the implementation of laws and systems, enhance family education, and foster a shift in public views.

The strengths of this study are as follows. This study used multi-stage stratified random sampling covering four regions and four levels of health care facilities in Hunan, ensuring good representativeness. Based on a validated WHO assessment tool, it combined on-site inspections, medical record reviews and caregiver interviews to comprehensively evaluate multi-dimensional primary care quality. Distinguishing sick and well children clearly revealed uneven clinical service quality, and mixed objective and subjective data reflected real service delivery.

This study has several limitations. First, the sample was limited to Hunan Province, so findings may not be generalizable to other regions. Second, caregiver interviews may have been subject to social desirability bias. Third, the study sample was based on data from 2021, during the COVID-19 outbreak. Restrictions and service adjustments introduced amid the pandemic inevitably influenced routine primary health care for children. Consequently, our results might cannot completely represent the post-pandemic service landscape, which may affect the generalizability of the conclusions. Fifth, the small sample size limited the feasibility of comparisons between different levels of facilities.

## Conclusion

Outpatient child health services in Hunan Province demonstrate strong performance in basic monitoring and free vaccine delivery, but face challenges in specialized sick child care, self-funded vaccine accessibility, and child protection. By strengthening policy oversight, expanding immunization coverage, and improving facility infrastructure, China can further enhance the quality and equity of child health services.

## Supplementary Information

Supplementary Material 1.

## Data Availability

The data that support the findings of this study are not openly available due to reasons of sensitivity and are available from the corresponding author upon reasonable request. Data are located in controlled access data storage at Department of Child Health Big Data Research Center, Capital Center for Children’s Health, Capital Medical University, Capital Institute of Pediatrics.
