# Supplementary material for: Quality assessment of primary health care for infants and young children in Hunan Province of China
Source: BMC Prim Care. 2026 Aug 10;27:402. doi: 10.1186/s12875-026-03516-x (PMC13737485; doi:10.1186/s12875-026-03516-x)
Supplement: Supplementary file 1 [file 12875_2026_3516_MOESM1_ESM.docx]

Figure S1 Rate of availability, storage conditions and validity of key medicines and supplies

| Medicine and supply type | Percentage medicine available and safely stored |
| --- | --- |
| Glucose solution | 97.1429 |
| Weighing scale – standing infants and children | 97.1429 |
| Antibiotics (e.g. Amoxicillin) for management of pneumonia | 94.2857 |
| Vitamin K | 91.4286 |
| Normal Saline IV | 91.4286 |
| Growth charts | 91.4286 |
| Bronchodilator (salbutamol) | 88.5714 |
| Functional autoclave or steam sterilizer | 88.5714 |
| Weighing scale (s) – non-standing infants | 88.5714 |
| Height length boards | 88.5714 |
| Vitamin A (100,000 IU and 200 000 IU) | 85.7143 |
| ORS | 82.8571 |
| Iron (Syrup) | 77.1429 |
| Inhaler/spacer for giving bronchodilator – sizes for different ages | 71.4286 |
| Hepatitis B vaccine | 68.5714 |
| Timing devices for counting respiratory rate (wall clock, other) | 68.5714 |
| Zinc drops/syrup | 65.7143 |
| Syringes for giving vaccinations | 65.7143 |
| BCG | 60 |
| Erythromycin/tetracycline eye ointment | 60 |
| Ringers Lactate IV | 57.1429 |
| Syringes for giving IM medicines | 57.1429 |
| Cups, spoons and water for giving ORS | 54.2857 |
| bOPV | 42.8571 |
| IPV | 42.8571 |
| Measles or MMR vaccine | 42.8571 |
| PCV | 37.1429 |
| Measles vaccine | 31.4286 |
| Mebendazole (500 mg) | 31.4286 |
| Pentavalent vaccine or individual vaccines for DTwP-HiB-HepB | 22.8571 |
| Ciprofloxacin or ceftriaxone for management of dysentery | 22.8571 |
| Micronutrient powders | 22.8571 |
| Therapeutic food formulations (RUTF) | 20 |
| Injectable Gentamycin for management of infant sepsis | 11.4286 |
| Co-trimoxazole (for prevention of complications due to HIV) | 11.4286 |
| ARV (HIV) | 8.57143 |

Figure S2. Policies, guidelines and standard operating procedures coverage for child health services at outpatient clinic

|  | Facility protocol coverage (%) | Staff oriented to policy percentage (%) |
| --- | --- | --- |
| Routine breastfeeding screening for infants 0–24 months (exclusive BF <6 months, | 97.1429 | 91.4286 |
| Routine health screening conducted for all children in outpatient clinics | 94.2857 | 91.4286 |
| Orders/regulations prohibiting use of infant formula and other linkages with milk formula companies | 94.2857 | 94.2857 |
| Clinical case-definition of anaemia | 91.4286 | 91.4286 |
| Definition of Severe Acute Malnutrition (SAM) and management protocol consistent with WHO standards | 88.5714 | 88.5714 |
| Definition of undernutrition and management protocol consistent with WHO standards | 88.5714 | 88.5714 |
| Routine complementary feeding screening and counselling for children 6–24 months | 88.5714 | 88.5714 |
| Definition of stunting and management protocol consistent with WHO standards | 88.5714 | 91.4286 |
| Protocol for feeding counselling for children identified with feeding problems | 85.7143 | 85.7143 |
| Accreditation and certification for the health facility includes child health standards | 77.1429 | 77.1429 |
| Treatment protocol for management of anaemia with iron and mebendazole consistent with WHO standards | 71.4286 | 71.4286 |
| Integrated Management of Newborn and Child Illness (IMNCI) standards adopted and used in outpatient clinics | 60 | 60 |

Figure S3. Infection control practices situation

|  | Protocol coverage rate (%) | Staff oriented to policy (%) |
| --- | --- | --- |
| Hand-washing practices | 100 | 100 |
| Use of double gloves for high risk cases | 97.1429 | 100 |
| Processing contaminated instruments | 97.1429 | 97.1429 |
| Disinfection of surfaces, floors and other items | 100 | 100 |
| Sharps and waste management | 100 | 100 |
| Disinfection of equipment including stethoscopes and thermometers between patients | 97.1429 | 97.1429 |

Figure S4. Maltreatment, injury and disability related protocol coverage rate

|  | Facility protocol coverage (%) | Staff oriented to policy (%) |
| --- | --- | --- |
| Protocols for routine developmental screening, management and referral for all children 0–36 months | 88.5714 | 88.5714 |
| Protocol for management and referral of children with suspicion of abuse or maltreatment at home | 28.5714 | 28.5714 |
| Age appropriate standards/ counselling guidelines for preventing childhood unintentional injuries | 77.1429 | 77.1429 |
| Age appropriate standards/ counselling guidelines for activities to stimulate or foster learning in the home | 88.5714 | 88.5714 |
| Disability-inclusion to support children or parents with disability to access the health care they require? | 68.5714 | 68.5714 |

Figure S5. Protocols and standards for screening and management of the sick child consistent with international/ WHO standards rate

|  | Protocol consisten with WHO standards rate (%) | Staff oriented to policy (%) |
| --- | --- | --- |
| Danger signs for immediate referral | 85.7143 | 85.7143 |
| Clinical assessment of dehydration | 85.7143 | 85.7143 |
| ORS and zinc for the management of diarrhoea | 85.7143 | 85.7143 |
| Assessment and management of bloody diarrhoea | 85.7143 | 85.7143 |
| Assessment and management of persistent diarrhoea | 85.7143 | 85.7143 |
| Clinical assessment of cough or difficult breathing | 85.7143 | 85.7143 |
| Definition of elevated respiratory rate for newborns and children | 85.7143 | 85.7143 |
| Assessment and management of wheezing | 85.7143 | 85.7143 |
| Clinical assessment of fever (stiff neck, malaria, where endemic, other bacterial causes) | 82.8571 | 82.8571 |
| Referral and pre-referral treatment of children with danger signs or very severe disease | 82.8571 | 82.8571 |
| Screening and referral of children with suspected TB or HIV | 80 | 80 |

Figure S6. Percentage of staff received skills training in the previous 5 years

Skills Training Topics

| Staff Type | IMCI | KMC/IYCF /Nutrition | HIV/TB | ECD /Childhood Disability | Injuries, abuse, maltreatment |
| --- | --- | --- | --- | --- | --- |
| Doctor | 47.9495 | 100 | 100 | 88.6435 | 81.0726 |
| Nurse | 60.8503 | 98.76 | 99.8229 | 91.9398 | 80.6909 |
| Technician | 52.0408 | 91.8367 | 100 | 90.8163 | 66.3265 |
| Pharmacist | 0 | 100 | 100 | 100 | 100 |
